# I’m shocked: informed consent in ECT and the phenomenological-self

**DOI:** 10.1186/s40504-018-0068-z

**Published:** 2018-02-13

**Authors:** Patrick Seniuk

**Affiliations:** 1 Södertörn University, Stockholm, Sweden; 2 Centre for Studies in Practical Knowledge, School of Culture and Education, Alfred Nobels allé 7, 141 81 Huddinge, Sweden

## Abstract

This paper argues that phenomenological insights regarding selfhood are relevant to the informed consent process in the treatment of depression using electro-convulsive therapy (ECT). One of the most significant side-effects associated with ECT is retrograde amnesia. Unfortunately, the current informed consent model does not adequately appreciate the full extent in which memory loss disturbs lived-experience. Through the philosophy of Merleau-Ponty, it is possible to appreciate the way in which memory loss affects a person’s self-experience, with emphasis given to one’s pre-reflective and embodied, relationship with things in the world. This paper aims to demonstrate that proper informed consent should acknowledge the extent to which repeated ECT treatments affect a patient’s sense self.

## Introduction

The sciences of both health and behavior continue to attract so-called “phenomenological research” into their orbit of praxis, and while we have good reason to welcome new avenues of inter-disciplinary research, we must also be cautious about stipulating what one can or cannot do with phenomenology.^1^ Unlike most appraisals of electroconvulsive therapy (ECT), this paper is not primarily concerned with statistically analyzing ECT data produced by research trails. Instead, using phenomenological philosophy as an analytical framework, my intention is to investigate the risks and harms associated with ECT, and the way patients are informed about the potential side effects. For, if psychiatric imperative is to treat “sick souls”, then clinicians would be remiss in over privileging a scientific attitude to the exclusion of all others. In particular, the main impetus for this discussion is precisely that an overly scientific attitude takes for granted that the depressed patient – the *soul* that is sick – is first and foremost a *self*. Accordingly, in addition to treatment benefits, a properly informed patient is a patient who not only “understands” the potential risks and harms of ECT, but who can also appreciate that the risks and harms will necessarily interfere with lived-experience, and by default, self-experience.

The 70-year history of ECT is marked by abuse and misuse. Much to the chagrin of contemporary psychiatric clinicians, the less than flattering aspects have been portrayed in narratives such as *The Bell Jar* and *One Flew Over the Cuckoo’s Nest*. These representations have, perhaps, encouraged beliefs that ECT is a cruel and anachronistic practice. Yet, for contemporary psychiatry, ECT is one of the four positively indicated treatment modalities to treat major depression. In fact, according to the American Psychiatric Association (APA), “electroconvulsive therapy has the highest rates of response and remission of any form of antidepressant treatment, with 70%–90% of those treated showing improvement” (APA 2010, 88).

Given the APA’s confidence in the efficacy of ECT treatment, as well as the World Health Organization’s projection that depression will soon be one of the most significant sources of disability globally (2017), it is curious that a treatment touted to be more effective than any antidepressant (Geddes et al., 2003). There are perhaps two obvious reasons why ECT remains contentious. First, most cases of depression are diagnosed and treated in primary care (Berrios and Callahan, 2004), which effectively limits the volume of patient contact with clinical psychiatry. Second, the APA’s enthusiasm for ECT has not imbued all researchers and psychiatrists with the same level of confidence. Despite the APA’s positive characterization of ECT treatment, use of ECT to treat major depression is disputed among professionals. Dissenters argue that the APA’s confidence in the efficacy of ECT is unwarranted (Read and Bentall, 2011; Johnstone 1999; Breeding, 2000). Advocates of the treatment, on the other hand, see little reason to doubt that the potential risks and harms associated with the treatment are acceptable relative to the positive treatment outcome (Fink 2001; Kellner et al., 2012).

The current informed consent model for ECT reflects an implicit commitment to the “brain-bound” model. With respect to potential side-effects, some of which include memory loss and cognitive impairment, it is common to find that they are characterized as predictable and reasonable outcomes that one would expect with direct brain intervention. These outcomes, however, are not considered from the perspective of the lived experience of the patient. To put it another way, the side effects are presented to patients such that they are characterized as factual probabilities. Likewise, the APA’s *Guideline Manual for ECT* expresses extraordinary high confidence in ECT treatment. Claiming that treatment is more successful than all first-generation anti-depressants, and that there are no immediate treatment contraindications (APA, 2008). When presented this way, the putative benefits of the treatment, in contrast to the harms, make the latter appear rather palatable.

Debate regarding the effectiveness of ECT treatment is divided and will likely remain unresolved for the foreseeable future. Then again, even if the treatment is proven to be effective beyond doubt, the problem regarding how clinicians ought to interpret the treatment’s potential risks and harms remains an open question. Understandably, advocates of ECT consider treatment side effects (risks) to be acceptable in proportion to the benefits. For instance, if one accepts that “ECT is a safe procedure,” and “there are no absolute contraindications for its use” (Sienaert 2011, 8), then it is unsurprising that clinical attitudes regarding risk and harm would be weighted in favor of the benefits. Yet, it is imperative that clinical support for ECT not cast a shadow over the risk/harm profile by extoling the treatment’s virtues.

In this paper, I focus on informed consent for ECT treatment and the way potential risks and harms are characterized for the patient. Using phenomenological philosophy, I highlight why it is misguided to view the post-procedure experience of retrograde amnesia as a limitation of function that is isolated to one dimension of a person’s life. I argue that the common experience of post-procedure retrograde amnesia will necessarily affect a patient’s sense of self. For depression patients who retain the capacity for autonomous decision-making, current bioethical attitudes about ECT and informed consent fail to adequately appreciate how “loss of memory” is interwoven with, and inextricable from, the patient’s experience of self. Furthermore, even if the treatment side effects are deemed acceptable by both patient and clinician alike—bearing in mind that full informed consent is not possible—I use phenomenology to highlight how informed consent procedures are not sufficiently nuanced to appreciate how side effects are implicated in the experience of suffering. When memory loss associated with ECT is presented as a discrete, abstract possibility (e.g. memory loss only inhibiting the ability to recall some information), it is taken for granted that side effects are, first and foremost, *lived* by a body-subject who is engaged and practically directed towards the world.

I will phenomenologically assess the side effects and harms associated with ECT in order to highlight that clinical use of informed consent, when applied instrumentally, fails to adequately prepare patients for the possibility that treatment side effects have implications for future lived experiences. I rely on the existential-phenomenological philosophy of Merleau-Ponty to illustrate how mainstream bioethics may be complimented by phenomenological insights. Ideally, successful integration of phenomenology with informed consent for ECT would encourage clinicians to appreciate how, for patients, side-effects are not merely statistical probabilities concerning functional values in one area of a person’s life, but are instead outcomes that interfere with one’s lived-experience.

A more robust informed consent process does not necessarily require a radical overhaul; the clinical reliance on an information “checklist,” undoubtedly has a role to play, but the role is limited. It is crucial to recognize that patients, while able to rationally “understand” memory loss is a potential harm, might fail to appreciate memory loss might interfere with everyday life.

From the phenomenological perspective, to characterize memory loss as a particular instance or isolated moment in which one simply cannot recall X, is to ignore that self-experience and memory are invariably woven together with one’s past, present and future; it takes for granted that existential modalities of perception, feeling, movement, and cognition do not constitute experience in the manner of part to whole. Just as the integrity of a fabric is compromised by one loose thread, lived experience is analogously vulnerable. Hence, in addition to providing patients with “information,” informed consent ought to be reinforced by the recognition that memory loss or amnesia will necessarily change the way a person is able to engage with his or her familiar or habitual life, as well as the general way in which he or she is directed toward the world. Risks and harms are more than just facts or factual probabilities. Having said this, it could be argued that the information provided to patients obviously *implies* that harms will limit their ability to continue with meaningful or important life projects. While there may be some sense in which this is accurate, “implicit” information is anathema to informed consent. Moreover, the informed consent process ought to unfold as a discussion between patient and clinician in such a way that the clinician brings to light possible consequences that might otherwise not appear salient to the patient.

## Why phenomenology?

With regard to depression, there may be a variety of reasons why phenomenological perspectives are only marginally influential in conversations about bioethical decision-making. Electro-convulsive therapy exemplifies psychiatry’s “brain-bound” interpretation of depression. According to this model, one’s “conscious mental states normally brought about through interaction with the world can be produced by direct stimulation of the brain and central nervous system” (Maiese 2015, 1). It makes sense, then, that life-world considerations would lack persuasive power in the domain of depression-related, ethical decision-making; phenomenal consciousness would be viewed to be the result of deeper-level, brain-related processes, which in turn means the patient’s experience of the world will not tell us anything significant about the neuro-biological dysfunction.

The “turn” toward phenomenological research has undoubtedly increased positive dialogue between the humanities and psychiatry. But phenomenology has become particularly salient for psychiatry because it is able to elicit information about the way ordinary life or lived-experience can be fundamentally disturbed, something that is outside of the operational criteria sets used in the DSM-5. Then again, the use of phenomenology in the health sciences often fails to deliver truly phenomenological research. Unfortunately, phenomenology is often mistaken to be synonymous with *experience*.^2^ Even if there is a weak sense in which this characterization is correct, it is nevertheless inaccurate. Phenomenology aims to uncover the invariant structures of experience through detailed descriptive analyses—those structures that cannot be understood via ground-up approaches (material) or top-down approaches (mind). Phenomenology provides a third option to access the nature of experience that these other levels of explanation take for granted. For example, “objective” descriptions that attempt to explain human experience fail to acknowledge the very source that makes objectivity possible: the world. A proper phenomenological analysis will be able to illuminate the way ECT side effects affect a patient’s I-world relation, which serves as the ultimate foundation for the possibility of self-conscious experience.

## Depression diagnosis: Then what?

The relationship between psychiatric diagnosis and psychiatric treatment is not always straightforward. Clinically, diagnosis and treatment must fulfill a logical relationship; treatment must be preceded by a set diagnosis. At the clinical level, psychiatric diagnosis of depression is guided by either the American Psychiatric Association’s (APA) *Diagnostic and Statistical Manual, fifth edition* (DSM-5) or the World Health Organization’s *International Classification of Diseases.* While both compendiums stipulate guidelines for diagnosis of psychiatric disorders, they do not provide therapeutic guidelines for subsequent treatment. Put another way, DSM-5 and ICD-10 may tell us what is wrong, but cannot tell us how to make it right. The diagnosis of depression, then, is necessary for treatment, but insufficient to determine the treatment itself. Unlike these two formal classification systems which are – more or less - globally recognized, treatment guidelines for syndromes such as depression are nationally heterogeneous. So, while DSM-5 and ICD-10 have made substantial inroads in reliably diagnosing syndromes such as depression, there is no international rubric to which could reliably guide clinicians to determine a course of treatment.

Neither the DSM-5 nor the ICD-10 is superior to the other, yet the relative differences between the classification systems prevents them from being used interchangeably:

we have two different and competing diagnostic systems: the *International Classification of Diseases* (ICD) and the *Diagnostic and Statistical Manual of Mental Disorders* (DSM). Both are useful lan- guages for communication – bridging the clinical/research interface and guiding treatment decisions. But both are also deeply flawed – unreliable in average everyday practice, largely unvalidated (sic), and lacking in biological tests {Frances, 2014 #805, 371}

Since there is clearly international disparity in treatment guidelines for depression, for the purpose of this paper I restrict my investigation to treatment guidelines stipulated by the American Psychiatric Association (APA). Even though the APA has achieved global reach with publications of DSM-III to DSM-5, the ICD-10 continues to be the classification scheme of choice for many European countries. But because the APA has published clinical treatment guideline manuals for depression, and serves a significant portion of the psychiatric profession, it seems prudent to begin the investigation with a concrete point of departure.

APA taskforces have been created with the sole purpose of establishing treatment guidelines for various categories of mental disorder. According to the APA’s *Practice Guideline for Treatment of Major Depression* (2010), there are four recommended treatment modalities for patients with mild to moderate symptom severity: psychopharmacological (anti-depressant), psychotherapy, combined pharmacotherapy and psychotherapy, or electroconvulsive therapy. Because all four treatment modalities are positively indicated, the guidelines state that treatment preference may be determined by patient preference. Still, many clinicians and researchers are dismayed by the inability to assuage the negative – if not paranoid – public attitude toward ECT treatment (Fink, 2009). Psychiatric professionals believe this attitude is unwarranted and ultimately leads patients to unnecessarily eschew an effective treatment modality for mild to serve cases of major depression (ibid).

## Electroconvulsive therapy

Contemporary ECT treatments are much more humane than earlier versions of the treatment. The medical procedure delivers electrical shocks through electrodes that are placed laterally on the exterior of the skull. To be effective, shock amplitudes must be strong enough to induce seizures in the brain. The stimulus may be transmitted unilaterally or bilaterally, depending on the psychiatrist’s prescription. Both types of treatment result in varying levels of cognitive side effects. Indication for stimulus strength is generally five times the seizure threshold in right-unilateral shocks, and brief pulse dosing of 50% of the seizure threshold during bilateral treatment (Shatzberg and Nemeroff 2009, 872). Patients are anesthetized and asleep during the procedure, which may be given up to three times per week, for a total of twenty treatments. Due to the use of anesthesia, the APA guidelines stipulate that patient information sheets should bring attention to the “remote possibility of death […] very low, about 1 in 13,000” (APA 2008, 320).

What the information sheet neglects to state is that the very low risk becomes much higher when the procedure is performed multiple times. The 1 in 13,000 risk of death for routine surgery requiring anesthesia is based on a one-time intervention. Bentall and Read (2010, 341) note that the use of sham ECT treatment as a control in ECT clinical trials is widely viewed to be too dangerous given the need for repeated bouts of general anesthesia. It is equally surprising that, despite the widespread acceptance of ECT, the mechanism of action remains unclear (Shatzberg and Nemeroff 2009, 862). Accordingly, potential treatment harms cannot be *absolutely* established.

## Informed consent

Properly informed consent must not only familiarize patients with the nature of procedure itself, but also provide adequate details regarding possible side effects. The essential function of informed consent is to disclose all relevant considerations associated with the procedure, so as to allow the patient to assert her right to self-determination (autonomy). The content of the disclosure should ideally be attuned to the individual patient’s life goals and projects, and the various ways a procedure could interfere with actualizing them.

The APA recommends that informed consent for ECT (2008) contain the following seven aspects:

1. description of the ECT procedure
2. why ECT is being recommended and by whom
3. applicable treatment alternatives
4. the likelihood and anticipated severity of major risks associated with the procedure, including mortality, adverse effects upon cardiovascular and central nervous systems, and common minor risks
5. description of behavioral restrictions that may be necessary during the pre-ECT evaluation period, the ECT course, and the recuperative interval
6. acknowledgement that consent for ECT is voluntary and can be withdrawn at any time
7. offer to answer questions regarding the recommended treatment at any time, and the name of whom to contact for such questions (ibid, 322)

The limited scope of this paper precludes an examination of the entire list, but it is clear that points 4) and 5) are interconnected and especially salient for the current discussion. A considerable obstacle to informed consent for ECT is establishing potential side-effect severity to be conveyed to the patient:

Choosing an appropriate informed consent process for ECT hinges on one’s interpretations of the scientific literature regarding the safety and effectiveness of this procedure. Different scholars, thus, are likely to have widely discrepant views concerning appropriate informed consent (Reisner 2003, 215).

Determination of the severity threshold depends on the clinician’s interpretation of the available side effect data, which in itself, seems perfunctory if it is not situated in the context of the patient’s present concerns, or what is meaningful for the patient in his or her “healthy” life.

Many patients seeking treatment (or those who are eligible) are not in extreme or debilitating situation; there lives are not totally devoid of meaning and are able to maintain a reasonably engaged life, albeit with varying levels of unpleasant feelings associated depression. Patients in such scenarios may be in a situation to evaluate more critically what, if any, changes to their lives they may be willing to accept. If a patient believes the risks are of little significance for her, then the treatment benefits have a significant (if not absolute) positive weight. But again, a patient’s ability to deliberate on risks, harms, and benefits, is dependent upon how this information is communicated. If memory loss affects one’s ability to actualize a certain way of life, then the suffering, which the patient sought to ameliorate in the first place, is merely displaced from one aspect of life to another, from the experience of depressed moods to gaps - perhaps significant gaps - in recent memories, for example. Over-confidence in treatment’s potential to relieve suffering associated with depression may fail appreciate the form suffering that might emerge from the side effects. So, while the depression symptoms may abate, a consequence of memory loss has the potential to interfere with a patient’s unreflective (non-thetic) engagement with certain life projects.

Consider a patient who, prior to depression, was devoted to violin playing as an amateur. To what extent, if at all, should this aspect of the patient’s life be incorporated into informed consent? If the potential for long-term memory impairment would significantly interfere with this aspect of her life—even if the side effect probability is low— would this consideration significantly change decision-making process concerning the suitability of ECT for that patient? It seems less relevant if we consider a counter example. If a professional violinist requires surgery on her wrist, and the procedure is otherwise considered low-risk for finger paralysis, in the context of the patient’s life, this risk becomes extremely significant. Even if the probability of paralysis is quite low, the patient’s livelihood is her profession, and without proper finger mobility her way of life is put at risk. Certainly, the reason for the surgical intervention is not unimportant. The surgery is indicated because it will benefit or improve her life in some way. Surgery to remove a malignant tumor is a situation that is considerably different from surgery for carpal tunnel syndrome. For the severely depressed patient (just like the case of a malignant tumor) ECT may viewed as a necessary intervention, and the imperative to sustain the patient’s life would supervene the risks. From a phenomenological perspective, in extreme cases of depression, the intentional structures that give rise to self-experience and intentionality would be weakened, if not altogether disturbed. So, in the extreme case, it would of little help to address risk and harm – with respect to the patient’s life-world – since the patient’s intentional horizons would already be significantly compromised, and her condition would be such that all her possibilities for action would be severely restricted. Either way, the conclusion is that in severe states of depression, informed consent for ECT many not be possible. From the mainstream bioethical perspective, the patient would lack the necessary autonomy that informed decision-making demands. From the phenomenological view, the extremely narrowed intentional state would not allow the patient to appreciate decision-making as one of her possibilities.

Notwithstanding the extreme situations where consent is not possible, to ensure a patient is properly informed, good clinical will require a treatment consultation, during which patients should be provided with a procedure information sheet. The APA (2008) recommends that clinicians adopt a version of their sample information sheet:

A common side effect of ECT is poor memory functioning. The degree of disruption of memory is likely to be related to the number of treatments given and their type. A smaller number of treatments is likely to produce less memory impairment than a larger number of treatments […] Shortly following a treatment, the problems with memory are most pronounced. As time from treatment increases, memory functioning improves. Shortly after the course of ECT, I may experience difficulties remembering events that happened before and while I received ECT. This spottiness in memory for past events may extend back to several months before I received ECT, and in rare instances, to one or two years. Many of these memories will return during the first several months following the ECT course. However, I may be left with some permanent gaps in memory, particularly for events that occurred close in time to the ECT course. In addition, for a short period following ECT, I may experience difficulty in learning and remembering new information (2008, 321).

The APA’s guidelines are not, prima facie*,* overtly contentious. And yet, the potential range of memory impairment listed in the guidelines is rather remarkable. Given the scope of the potential wide-ranging side effects, when the potential risks associated with ECT are presented matter-of-factly, rather than establishing clarity, there is the potential for opacity. A number of questions should be raised about ECT’s side effects, as stated in the quote. For example: in what way do all these possibilities for memory loss relate to one another? Are these possibilities mutually exclusive? Do they supervene on one another? The listed possible memory impairments are ambiguous, especially insofar as it is unclear how a patient’s memory might be compromised as a whole over time. The effects range from some temporary spottiness and gaps to *permanent* gaps and *permanent* impairment in remembering. The APA taskforce on ECT (2001) is somewhat less equivocal about the possibility of memory impairment:

Retrograde amnesia occurs *to some extent in almost all ECT recipients*, […] In some patients the recovery from retrograde amnesia will be incomplete, and evidence has shown that ECT can result in persistent or permanent memory loss (APA 2001, 71 emphasis added).

Contrast this with the lengthy APA treatment recommendation, which claims that “a common side effect of ECT is poor memory functioning…,” and a discrepancy between the two formulations is clearly recognizable. This discrepancy cannot be reduced to a matter of semantics. In medicine, the difference between “common” in patients and present in “almost all” patients is not qualitatively insignificant. Moreover, it is not clinically insignificant that the APA treatment recommendations adopt the weaker position, whereas it is the APA taskforce that further emphasizes the risk of memory loss. The purpose of informed consent is – at least in part - to acknowledge the power imbalance between the clinician and patient. If the restricted scope of disclosure in treatment recommendations is taken as a benchmark, then the clinician’s duty to disclose anything beyond this, anything beyond this is superogatory. Informed consent is indeed a balancing act of judgment However, because the clinician holds a privileged epistemic position in relation to the patient, the idiomatic “less is more,” is not absolutely defensible.

## Harm & Severity

When trying to understand what constitutes a potential risk for patients in ECT treatment (or non-treatment), clinicians reach “the point where the views on ECT among psychiatrists [have] become polarized and examining the literature in detail does not conclusively move one beyond this impasse” (Stefanazzi 2013, 86). This said, despite the professional polarization, proponents and detractors alike derive their conclusions from within the “objective attitude” rather than from a phenomenological attitude. The basis for disagreement concerning ECT is descriptive, meaning that facts are the point of contention. This dispute takes place within what Merleau-Ponty calls “the objective thought of common sense and of science – which in the end makes us lose contact with *the perceptual experience of which it is nevertheless the result*” (2012, 74, emphasis added). Proponents and opponents alike take for granted that empirical facts related to risk, harm, and benefit, only become meaningful based on pre-reflective or lived experience. Put another way, the statistical expression of potential of memory loss presupposes that we already have an experiential acquaintance with memory. To treat memory as an object to be studied presupposes that one is already acquainted with an experience of memory. Of course, the claim is not that subjective experience of memory has veridical superiority over an objective description of memory, per se. What pre-reflective experience *does* provide is a measure of reference to which we can appeal when assessing whether empirical descriptions of memory are sound. Lived-experience can confirm or over-rule empirical evidence, and vice versa.

Nevertheless, even assuming that we could achieve (near) unanimous clinical consensus regarding how to interpret the severity of risks and harms, it should lead us to conclude that the informed consent process for ECT need only ensure patients are provided with a comprehensive list of potential side effects. Stipulating that “memory loss” or “amnesia” is a possible result of the procedure is nothing more than an abstraction. The burden on clinicians, as I will later show, is to clarify the extent to which a person’s life may be impacted by this procedure. As Robertson and Pryor wrote, “If the term anterograde amnesia must be used, it should be clearly defined as difficulties with memory in daily life, [with] examples given” (2006, 229).

Once we acknowledge that side effects are not simply self-contained, physical bi-products, the possibility of a more accurate, phenomenologically inspired consent process becomes conceivable. Unless side effects are incorporated into the patient’s meaning horizon, a structure that guides her comportment towards the world without the necessity of explicit reflection, then procedures for informed consent will remain impotent.

It is clear that critical approaches to informed consent need not adopt phenomenological frameworks. As we saw earlier, there are plenty of critics who share, at least in principle, the concern that patients must be informed of the way side effects will bear on their life. Despite this sensitivity, I will show how phenomenological insight offers something unique. In particular it reveals the way existing informed consent processes do not take into how patients’ I-world relations are interrupted by ECT. Among other things, these relations include a patient’s temporal orientation to the past and the future, which is clearly relevant to memory. The model of informed consent that I have outlined until now imagines side effects discretely, as an object inside me that bear no reference to the way they manifest as expressions of self (or person). In other words, standard informed consent procedures fail to account for the way ECT treatment will impact the global nature of patients’ self-experiences in everyday situations.

In everyday life we are oriented toward the world through situations we find ourselves in. Based on previous experience, we learn to deal with the various situations that matter to us or call for some sort of resolution. Under “normal” circumstances we seamlessly engage with our situations according to habits that we have acquired through similar situations that we have successfully “figured out.” These habits become embodied as repertories or bodily “know how”, which we, in turn, reatain as “sedimented” or embodied capacities for acting according to various situational demands without explicit knowledge of doing so. Unlike “unconscious” behavior, which is by definition, inaccessible to conscious thought, our familiar behaviors that take shape across perceptual and bodily space are pre-reflective; they make reflective experience possible, for without the pre-reflective modality, the act of reflection would have nothing to turn itself toward. Importantly, our familiar and pre-reflective experiences entail more than we could ever possibly articulate. Some aspects of my experience are not as salient as others, yet the aspects that remain at the periphery of my experience are, nevertheless, co-present as a background that allows me to focus in on some thing particular, or the ability to turn my attention from one thing to another.

When I examine the book on the table, the book stands out for me by virtue of the table as a background for the book as a figure. In the same way, my past experiences, as well as potential future experiences, structure my everyday life (i.e. perceptions, movements, feelings) even though I am not necessarily explicitly aware of them. To be in a concrete situation is to also be unreflectively engaged with the world under open horizons that offer possibilities for future experiences. “My present perception,” writes Eric Matthews, “is not something separate from past perceptions, to be causally affected by them, but part along with them of a continuing life history” (2002, 60). My life goals and projects are “haunted” by what has come before and what is yet to come. Everyday life is bordered by possibilities or potentials that I am aware of, but only vaguely:

The landscape I have now before my eyes can certainly announce to me the shape of the landscape hidden behind the hill, but it only does so with a certain degree of indetermination, for here there are fields, while over there might be a forest, and, in any case, beyond the next horizon I know only that there will be either land or sea, and beyond again, either open sea or frozen sea… I know only that there will be something to see in general. I possess no more than the abstract style of these relationships (Merleau-Ponty 2012, 346)

An horizon denotes an opening onto the world which discloses what actions are available to me as a body-subject. It is important that we do not restrict the notion of horizon to only the future. It is reciprocally a relation to the past. My behaviors in a particular situation will open up other situations towards which I may turn, but this capacity is the result of having encountered similar situations. We can say that our past serves as a sort of scaffolding for the future, which is built-up in the present. Thus, our lives are invariably shaped by, amongst other things, memory. Yet, memory in the traditional sense, as an object that is identified in the brain, does not square well with the phenomenological description of an embodied subject’s relation to the world. To fully grasp the implications of memory loss associated with ECT, we need to consider a phenomenological account of memory and the relevance.

## It’s me, remember?

I have chosen to focus on the potential risk of memory loss associated with ECT treatment because I want to illustrate how memory is phenomenologically significant with respect to the experience of selfhood. To be clear, the reference to “self” denotes a phenomenon that is not to be understood as a discrete entity or kernel located somewhere *in* a person, as such. In classic “psychological” approaches to self, memory is often considered a primary trait (Locke 1836; Parfit 1984). I will illustrate how a phenomenological approach to self may also place significance on the role of memory without reducing the latter to the former. However, despite the superficial accordance between the two, what makes the phenomenological approach fundamentally unique is the embodied nature of memory in selfhood.

Both Michel Henry (1975) and Merleau-Ponty (2012) argue that being-in-the-world (or intentionality) arises only by virtue of being a body. As a body, we inhabit the world, where *in-habit* takes on a literal sense: our bodily habits are expressions of who we are. For Henry in particular, the body is, itself, memory. Our bodily capacities and expressions articulate bodily knowledge, which is established through previous experience. In the action of reaching for and grasping a glass, I do so because the situation solicits the acts of reaching and grasping in the form of bodily remembering. That is, my ability to grasp is predicated on a pre-understanding of “grasping,” a non-conceptual form of knowledge elicited by the practical demands of a situation without a need to invoke propositional content.

Despite the emphasis I have placed on memory, it be incorrect to assume, Merleau-Ponty’s epistemology is over-determined by memory. For instance, in *Phenomenology of Perception*, he describes how an organist, after an hour of practice, is able to perform on an unfamiliar organ. It is not an instantiation of memory, but rather, “he sizes up the instrument with his body, he incorporates its directions and dimensions, and he settle into the organ as one settles into a house” (Merleau-Ponty 2012, 146). Non-conceptual knowledge is ultimately interwoven with bodily space, movement, and perception. Even though memory may engender us with a sense of situational familiarity, it does not fully determine how we take up the situation we find ourselves in presently.

The crucial point for this paper is that memory must be viewed as embodied, rather than rooted somewhere in the brain. From the phenomenological perspective, though memory need not be limited solely to bodily action, the act of remembering or recalling is predicated on being embodied. To remember is not to re-present or to picture previous experience in the form of a mental picture. It is an intentional act that summons, or following Husserl, re-enacts, a past experience (2012, §45). Whether or not a particular memory is elicited spontaneously or by prompt, the re-enacted experience *necessarily* draws upon the original embodied act. The embodied experience is a necessary requirement. However, is it a *sufficient* requirement?

Types of memory, such as short-term memory, long-term memory, declarative, procedural, and so on, belong to the category of “objective thought” (Merleau-Ponty 2012). These modes of memory are almost exclusively cognitive, and may be called “bodily” only in the weak sense that the materiality of the brain is “bodily.” Following this line of thought, we must clarify: *where* and *how* is the memory of objective-thought “stored”?

One of the most basic phenomenological tenets of intentional consciousness espouses that consciousness is not a container for the mind or for mental content. Rather, consciousness is an act or a type of experience conceivable only against the background of the world. If the seat of memory is located in the brain, the burden of proof remains with the empirical sciences to account for the metaphysical status of memory and mental content. Even with the formidable range of neuro-imaging now available to researchers, we must tread with caution when studying the “inner workings” of memory. Neural activity images recorded during acts of remembering and the like should not be viewed as indexical of memory’s presence.

There is no analogy between pointing to the prefrontal cortex to locate the presence of memory and pointing to a computer electronics board to indicate the storage place of data*.* Moving from the natural attitude (everyday, commonsense) to a phenomenological one, the brain and its processes are not viewed as the locus of memory. Rather than focusing on the body-subject’s internal workings, memory is sought in its relation to the external world with which intentional consciousness is inextricably interwoven. I think Mazis captures the importance of this connection well, when he writes that “both memory and self [are] more like a felt force or like a certain way or style *enmeshed in the way our world comes forth to us*, yet not something that could be grasped or even seen directly” (2015, 52, emphasis added). To a large extent, then, the self is something that resists articulation. It is present as a felt experience and manifested as a general outline (or physiognomy) that is open to and affected by the contingencies of the world.

Merleau-Ponty is well known for his case study of the patient “Schneider” adopted from the psychologist, and Merleau-Ponty’s contemporary, Goldstein. Schneider was a patient who suffered a shrapnel injury to his skull, which subsequently impaired various cognitive and personal functions. Merleau-Ponty’s account is seminal because his analysis highlights how Schneider’s symptoms are neither visual dysfunctions nor the result of brain lesions, as such. Instead, his analysis establishes that the source of Schneider’s problems—such as abstract movement or using imagination—is a disruption in body movement (motor-intentionality). Put another way, the patient was unable to place himself in imaginary situations. He was able to deal exclusively with objects placed in front of him, which he could do only through thematization of his body. This analysis demonstrates that disorders and dysfunction are not necessarily explained by appeals to common sense.

A possible objection to what I have outlined might be that I have simply recognized a distinction between two modes of memory: implicit memory and explicit memory. Perhaps intellectual memory serves some purposes and bodily memory serves others. Here, implicit memory would be exemplified by our capacity for unreflective bodily action (e.g., that which governs practical knowledge, or know-how of walking, grasping, etc.), whereas explicit memory is a feature of imaginative or cognitive processes, such as describing a past experience (e.g., when we say to the best of your recollection, or recall a time when…).

Though this objection has an intuitive appeal, it relies on the assumption that a there exists a difference in kind between explicit memory (or intellectual memory) and implicit memory (bodily memory). If we maintain the phenomenological attitude toward memory, then we must eschew claims that memory is “located” or stored in distinctly different forms. Since an act of recollection is the renewal of a past experience, all recollection is necessarily anchored in the world and body dyad. No experience is possible without the body, which implies that all recollection renews a prior experience that always, in some way, originates through bodily *responsiveness* to the particular demands of a situation. A child who must memorize multiplication tables for a school test offers an example of a situation that might take on different senses. It could provoke anxiety for fear of failing a test; subsequent disappointment of parents; feelings of inadequacy; frustration from being unable to understand; and so on.

Temptation to explain this example by resorting to folk psychology concepts such as associationism must be rejected. The student’s anxiety does not arise as a result of linking disappointed parents with failing math. On the contrary, consider the following from Merleau-Ponty, who says, “we believe that our past, for ourselves, reduces to the explicit memories that we can contemplate” (2012, 413). Merleau-Ponty insists that memory is not a matter of performing intellectual gymnastics. That I become anxious upon hearing of a math test reflects causal explanations that are common to folk-psychology or those of psychologism. What we discover when completing a phenomenological analysis is that:

To remember is not to bring back before the gaze of consciousness a self-subsistent picture of the past, it is to plunge into the horizon of the past and gradually to unfold tightly packed perspectives until the experiences that it summarizes are as if lived anew in their own temporal place. (Merleau-Ponty 2012, 23).

Adopting this non-representational interpretation of memory, whether we are warranted to claim that memory is exclusively the outcome of *internal* processes is open to contestation. Humans are fundamentally directed toward the world in a purposeful way. We are literally “caught-up” in and amongst things, even though we are rarely cognizant of the many ways we habitually deal with our surroundings. Once, however, the phenomenological perspective discloses our habitual engagement with the world, it also becomes clear that we are not simply *in* the world, in this very spatio-temporal location. Instead, following Merleau-Ponty, “we must not say that our body is *in* space, nor for that matter *in* time. It *inhabits* space and time (2012, 140, emphasis original).”

## It’s okay to be self-ish

Despite lacking concreteness, the self is much more than an enduring material body or a psychological continuity over time. The formation of this elusive phenomenon is bound by things we encounter and re-encounter in the world, a style of comportment towards both people and objects. Through our experience of the world, we are brought to ourselves. Jacobson rightly notes that self “is seamless in my experience. It is not me alone who holds this self together; rather, things and my world hold me” (2015, 37). Memory is a crucial feature of a phenomenological characterization of self. The essential connection between memory and the past means that temporality is somehow integral to the nature of selfhood.

Intentional consciousness is spread among things in the world. Contact with the otherness of the world reflects back to us something of ourselves. Jacobson writes:

Being forcibly brought back to my history by a place or another person challenges this notion of memory as first and foremost belonging to me. There is perhaps more sense in saying that we belong to our memories, that memory, in other words, provides the home in which we *can* be and become ourselves (2015, 31, emphasis original).

In a sense, memory allows for the possibility of feeling-at-home in our habits. When our habitual way of engagement with the world is disrupted, we become conspicuous to ourselves. Suddenly, we are expressly aware of being the *I* of our I-world relation. World disruption is experienced as a rupture with operative intentionality, with our habitual interaction with the world, which I believe is exemplified nowhere better than depression (and illness in general). In every day life, the world is the background upon which we are able to move, perceive, feel, and think. In “normal” life, the world usually taken for granted, as is the case with figure-background structures. The background must remain inconspicuous in order for us to appreciate the foreground, though its presence must never disappear altogether.

The strong feelings associated with depression can make bodily experience become the central focus of experience as a whole. Not only do the strong, diffuse feelings of suffering make one aware of oneself, the world is, in turn, experienced anomalously; favorite objects or activities no longer solicit the same feelings they once did, foods taste bland, people feel distant or hard to connect with, and, very often, depression is accompanied by a strong sense of being detached from the world. Hence, to some degree, patients who receive ECT treatment likely already experience disturbed habits or world-relations. The question, then, is whether or not the amnesiac side effects of ECT treatment exacerbate or merely trade-off on I-world disturbances.

## Do you remember that time...?

If we trace the intimate relationship between self, temporality, and memory, through Merleau-Ponty’s ontology, we quickly find that the common sense understanding of the past misconstrues the structure of temporality. The past is not a static thing that is forever inaccessible to us. Connection with the present is not ruptured, and it is not possible to project a future without the presence of the past. We encounter the past, then, in the presence of absence. Our bodily habits express a connection to our past, which we have incorporated in the form of bodily norms (Merleau-Ponty 2012). These norms are perhaps most often disclosed through neurotic expressions or behaviors. Certain manners or styles of dealing with situations are established through our past experiences, the results of which give our life in the present a “flavor” of the past whilst remaining past.

The past is always situated in our present, just as our future springs up from the present. It is never fully closed off or fully open. To use a simple illustration, our past guides our present irrespective of whether we make this connection explicit. “Memory proper,” say Morris and Maclaren, “makes the past stand out as distinct from the present it steers” (2015, 8). This does *not* mean, however, that our past determinately fixes our future; were that the case, the past would, effectively, disappear. It would no longer be present. We might imagine the following analogy: a sailboat is steered by its rudder, but it also relies on the sails, the ropes, and various combinations of ways in which these components work together. But *without* the rudder, the fate of the sailboat is given over to the natural elements; the ship becomes determined by external circumstances.

The self is a fuzzy notion insofar as it is determined yet in-determinate. To exist is to be inexorably bound to a past that is founded through intentional contact with the world. Because we are in the world perceptually and bodily, the sedimented habits of the past structure our present expressions of self. Put in another way, similar to the pattern of a wave, the self goes out toward the world and, as if cresting, gathers itself back together with all it has collected; we go out toward the world with certain bodily capacities and return to ourselves with new motor experiences, affective experiences, perceptual experiences, and cognitive-linguistic experiences taken from the world. As Mazis says, “the self, then, is not something that we possess or that appears with clarity, but in its enmeshment with the world is difficult to discern” (2015, 50).

## Bringing it together

Some readers may consider the above discussion concerning selfhood too abstract to be applicable to the health sciences. However, I believe that phenomenology has practical relevance for bioethical questions related to ECT. Minimally, phenomenology illustrates that informed consent for ECT will always be insufficient if we continue to frame that consent in terms of commonly taught ethical dilemmas in medicine. Applying bioethical principles to exemplar cases of ECT treatments distorts the nature of both depression and selfhood. Approaching the ECT informed consent procedure as an exercise of utility (risks vs. benefits) obscures the way ECT intervention ought to be assessed, namely how the procedure might affect the everyday, habitually engaged self.

It must be acknowledged that ECT is indicated for treatment of depression in cases of extreme patient suffering. Such patients are near-catatonic or otherwise unable to care for themselves. When measured against this symptomatic standard, my considerations regarding selfhood are generally inapplicble. However, this is because truly severe depression precludes the patient’s ability to understand the nature of any procedure. In this case, then, informed consent is no longer possible. When a patient is deemed to lack the capacity for autonomous decision-making, the psychiatrist (or psychiatry team) reasons that the potential benefits of ECT supersede its risks. In other words, it is highly unlikely that the outcome of the treatment will make the situation worse than it is. In the face of the extreme suffering manifested in depression, the argument for ECT is a forceful one. But what of depression that’s is not catatonic, or that incapacitates? Or what of the patient who has not *always been* in a catatonic state?

We need to address the group of patients who have the ability to exercise his or her will, or who at least demonstrate the a functional ability to care for themselves, even if it is greatly reduced. The guidelines for determining who is and is not eligible for ECT are extremely porous. Depression that is not incapacitating, but unresponsive to other treatments, positively indicates ECT. If a patient is autonomous and is offered ECT treatment, then my argument—that informed consent ought to incorporate phenomenological reference to selfhood—is relevant. The suffering experienced in depression is related to, or characterized as, a disturbance of self (Karp 1996; Svenaeus 2014; Ratcliffe 2015). Therefore, when ECT is prescribed to treat less severe cases of depression, proper informed consent must articulate that the side effects may include a sense of suffering not dissimilar from that of depression itself.

We can draw parallels to the problem of psychopharmacological treatment of depression. Patients are often reluctant to begin treatment course of anti-depressants for the very reason that the treatment side effects often (and indirectly) lead to mental or physical states that are intolerable for the patient (e.g. weight gain, decreased libido, flattened affect), thereby leading to further feelings of depression. Pestello and Davis-Burman, who studied attitudes of persons using anti-depressants, conclude that:

treatment was often [viewed to be] worse than the illness… [The descriptions] refer to intense physical struggles that certainly disrupt functioning, which in turn impacts the way posters feel about themselves, and the medication they are taking. Regardless of any positive impact that anti-depressants might have on one's depression and ability to cope, [online forum] posters talked about feeling like a different person because of the number of physical side effects (2008, 353-54).

What is most striking about their finding is the relation between disrupted function and self-feeling. It exemplifies the way un-reflective or every day life becomes inhibited, not only on account of physical changes, but also in the way the patient feels about herself. It is also illustrates why clinicians need to assess the risks and harms of the intervention in question. However, the crux of the matter lay in how side effects are interpreted. For instance, higher incidence of irritable bowls associated with anti-depressants is never *just* irritable bowls; it is the total way the patient must direct herself toward the world, which might include not wanting to go places without full knowledge of what washroom facilities are available and the relative distances to one’s destination. In the extreme case, it could mean not wanting to leave the house at all.

Under what circumstances might voluntary consent be obtained when the potential risk of short-term (and sometimes long-term) amnesia exists? Having explored a basic phenomenology of memory, it seems evident that informed consent for ECT isolates memory as an effect rather than an essential structure of the patient’s life; it is *mere* memory. Yet, good clinical practice should always consider the importance of a patient’s life projects, and how treatment could impinge upon them. Memory loss can disrupt both one’s sense of self and life projects.

Of course, we must acknowledge that not all ECT outcomes are negative or experienced negatively by patients. On the whole, the data are inconclusive. When asked about the treatment, one patient reports that it “‘must have done some good for me. .. because I’m not as paranoid as I was. .. I ‘m a bit more normal… it really brought me back to reality” (Koopowitz et al. 2003, 52). By contrast, another patient who reported having paranoia pre-procedure found that it was exacerbated after the therapy: “‘I don't believe that I can speak as coherently—I don't think my train of thought is connected. I am more apprehensive. I am more fearful at... what will happen to me’” (Warren 1988, 289). One patient reported that she felt more like herself (Koopowitz et al. 2003), while yet another patient stated that she had the experience of no longer knowing who she was (ibid.).

There is one report in particular that is especially salient from a phenomenological perspective. The case highlights the phenomenological worries raised about memory loss and selfhood due to ECT. A woman who had been interviewed for research regarding patient experiences of ECT described how, after treatment, she was unable to remember that she gave birth to a child nine months earlier. After being reminded of the child’s existence:

She appeared to have lost her affective memory of him as her child: (Shirley Arlen) ‘I guess I feel sort of strange with him. In being with him. I don't know, I guess I just feel sort of strange with him… I just don't even feel like he's mine, for some reason... I think he's nine months ...I really don't know. I can’t remember when he was born’ (Warren 1988, 295).

Not only does the patient experience the loss of conventional memory attributed to “objective thought,” her description also captures the way in which the intentional threads that draw her toward the world through bodily and affective feelings have slackened. The “invisible” intentional threads that previously put her in contact with the world, a world that is otherwise pregnant with significance or affective salience, now fail to make contact. Thus, while the aim of ECT treatment is to reduce patient suffering, experiences like the one noted above suggest that ECT treatment carries with it a risk of exacerbating the suffering of depression.

My final considerations concerning ECT and informed consent are not necessarily concerns restricted to the phenomenologist. For instance, it remains unclear that patients understand what they are consenting to when they decide to undergo ECT; is the memory loss itself the mechanism that makes the patient feel better? The nature of depression may be such that the patient will consent *because* they desire memory loss. Reflecting on the following patient descriptions, is it viable to continue thinking of the informed consent process for ECT as properly informed without establishing a reference to selfhood?

‘I felt as though I had become a completely different person […] And some positive things did come out of it because I went out and I worked for a year and I was discharged from hospital. It was at a very high cost, obviously. You feel you’ve got to adapt to this new person that you are. For a year or two afterwards I felt very mad. I felt I’ d lost the person I used to be’ (Johnstone 1999, 86)

Or, consider the following: “‘It happens all the time. It’ s tiny little things, which on their own don’ t really matter, but it’ s this permanent sense of something that you’ve lost’”(ibid). In addition to memory proper, at the heart of many ECT side effects is a disruption to intentionality. Though intentionality may be disturbed in various ways, it nevertheless appears to be the case that for some patients:

The commonest complaints were inability to follow films, books or TV programmes, and problems with facial recognition. These disabilities were both frustrating and embarrassing. Less tangible was the general loss of sense of self described by a few participants (ibid).

The descriptions reflect not just intentional disturbances, but also motor-intentional disturbances. Problems reading books, watching films, and recognizing faces are not simply attention deficits. These skills are related to “seeing”, which we accomplish with our eyes—not as stimuli receptors, but as a capacity to move across the surfaces of objects in our visual field. Lack of facial recognition in particular is tied to an inability to grasp a whole. It is not a mis-recognition of individual parts, such as nose, ears, eyes, etc. In fact, if we were shown only the individual parts of a face, it is unlikely we would be able to identify the person. What we grasp is the way these parts “hang” together to provide a general outline—something we do not perceive as sense data, but by recognizing a certain physiognomy.

Having illuminated some of the problems associated with ECT according to first-person accounts, it is clear that the current ethos regarding informed consent does not adequately entertain meaningful considerations for the patient. It is self-evidently unhelpful for patients to be told that they might experience intentional disturbances to intentionality. Technical language, whether scientific of philosophical, is likely to obfuscate a situation that demands clarity. Nonetheless, I do think that patients should be presented with the possibility of experiencing existential changes. Memory is not a sufficient feature of selfhood. Yet, it is clearly one of necessity. It is equally clear that disturbances to memory can be profoundly unsettling experiences that may disrupt how we do or do not feel at home in the world.

## Conclusion

The ambiguity of ECT’s efficacy and its side effect profile means that the treatment outcomes have potential consequences for the experience of suffering in depression. For cases where it is possible to solicit informed consent, I have argued that potential disturbances in the relationship between memory and sense of self is not unimportant information for the patient if we believe that the standard informed consent process presents risks and harms as abstractions. If patients are given the option of ECT, the likelihood that depression symptoms will remit needs to be properly considered against the possibility that ECT treatment may compromise the patient’s post-procedure self-experience. Prevailing attitudes interpret memory as a receptacle. When memory function is impaired, the impairment is localized. However, from a phenomenological perspective, our memory is more than a catalogue collection of discrete experiences of time past. Memory is inextricably bound up with our past and future sense of self, not to mention our bodily habits and world-directed engagement. Hence, if phenomenological insights remain excluded from bioethical decisions concerning ECT and depression, the treatment will remain divisive. More importantly, the phenomenological discussion of self is inline with the patient-centered approach that has become fashionable in many of the heath professions. The insights I have highlighted enable clinician’s to empower patients with a sense of control. It is a form of hospitality that, even if the clinician finds the patient difficult or unruly, lets the clinician demonstrate that they understand the patient beyond the confines of the psychiatric setting. The model of informed consent I have discussed represents the clinician as someone who makes everything he or she has at her disposal open to the patient. The specialized form of knowledge with which clinicians are endowed, is one obstacle to proper informed consent. One way to neutralize this obstacle is to place some sense of power back toward the patient. No list of qualities or personal traits could ever tell us who or what a patient is, or is not. A person is the entire way he or she relates to, or is an expression of, her relation to the world, through what Merleau-Ponty calls a style. If we acknowledge that the potential side effects of ECT, when taken in isolation, fail to describe how they may affect the patient, and that the sense of self entails more than one could ever expressively exhaust, then clinicians may be prompted to re-assess their views regarding the potential risks and harms of ECT with the appreciation that a patient is not just a person, but self whose life always projects more than what we see.

## Footnotes

1 See Stella Sandford {, 2016 #771}for an excellent discussion on some of the problems that arise when researchers attempt to integrate phenomenological philosophy and qualitative studies.

2 For instance, the DSM has long purported to be “phenomenological” given that it *describes* signs and symptoms based on patient observation.
